# Joint association of smoking and physical activity with mortality in elderly hypertensive patients: A Chinese population-based cohort study in 2007–2018

**DOI:** 10.3389/fpubh.2022.1005260

**Published:** 2022-09-29

**Authors:** Yating Yang, Huilin Xu, Xiaoqin Liu, Jiong Li, Zeyan Liew, Xing Liu, Chen Huang, Jingjing Zhu, Jinling Zhang, Linli Chen, Yuantao Hao, Guoyou Qin, Yongfu Yu

**Affiliations:** ^1^Department of Biostatistics, School of Public Health, The Key Laboratory of Public Health Safety of Ministry of Education, Fudan University, Shanghai, China; ^2^Shanghai Minhang Center for Disease Control and Prevention, Shanghai, China; ^3^NCRR-National Centre for Register-Based Research, Aarhus University, Aarhus, Denmark; ^4^Department of Clinical Medicine, Aarhus University, Aarhus, Denmark; ^5^Department of Clinical Epidemiology, Aarhus University, Aarhus, Denmark; ^6^Department of Environmental Health Sciences, Yale School of Public Health, New Haven, CT, United States; ^7^Department of Epidemiology, School of Public Health, The Key Laboratory of Public Health Safety of Ministry of Education, Fudan University, Shanghai, China; ^8^Department of Medical Statistics and Epidemiology, School of Public Health, Sun Yat-sen University, Guangzhou, China; ^9^Shanghai Institute of Infectious Disease and Biosecurity, Shanghai, China

**Keywords:** hypertension, cardiovascular disease, all-cause mortality, physical activity, smoking, interaction, elderly

## Abstract

**Background:**

Although associations of physical activity and smoking with mortality have been well-established, the joint impact of physical activity and smoking on premature mortality among elderly hypertensive population was still unclear. This study aimed to assess association of physical activity, smoking, and their interaction with all-cause and cardiovascular disease (CVD) mortality risk in elderly hypertensive patients.

**Methods:**

We included 125,978 Chinese hypertensive patients aged 60–85 years [mean (SD) age, 70.5 (6.9) years] who had records in electronic health information system of Minhang District of Shanghai, China in 2007–2015. Cox regression was used to estimate individual and joint association of smoking and physical activity on all-cause and CVD mortality. Interactions were measured both additively and multiplicatively. Additive interaction was evaluated by relative excess risk due to interaction (RERI), attributable proportion due to interaction (AP) and synergy index (S).

**Results:**

Among 125,978 elderly hypertensive patients (median age 70.1), 28,250 deaths from all causes and 13,164 deaths from CVD were observed during the follow-up up to 11 years. There was an additive interaction between smoking and physical inactivity [RERI: all-cause 0.19 (95% CI: 0.04–0.34), CVD 0.28 (0.06–0.50); AP: all-cause 0.09 (0.02–0.16), CVD 0.14 (0.04–0.23); S: all-cause 1.21 (1.04–1.42), CVD 1.36 (1.06–1.75)], while the concurrence of both risk factors was associated with more than 2-fold risk of death [hazard ratio (HR): all-cause 2.10 (1.99–2.21), CVD 2.19 (2.02–2.38)].

**Conclusion:**

Our study suggested that smoking and physical inactivity together may have amplified association on premature death compared to the sum of their individual associations, highlighting the importance of improving behavioral factors in combination and promoting a comprehensive healthy lifestyle in hypertensive elderly.

## Introduction

Worldwide, there were 703 million people aged 65 years and above in 2019, comprising 9% of total population (1); by 2050, those numbers are expected to rise to 20% (1.6 billion) (2). Among the elderly, hypertension is the leading cause of premature death and cardiovascular disease (CVD) globally (3). Approximately 31.1% of adults worldwide live with hypertension, and 9.4 million people die from hypertension and complications every year (4). While hypertension is more prevalent among the elderly (5, 6), its treatment and control have been suboptimal (3). For example, in China, the prevalence of hypertension in people over 65 years old is as high as 58%, while only half of them receive antihypertensive medication treatment and the control rate is less than one-fifth (7). With the increasing life expectancy and aging trend of the population (1), the burden of disease from hypertension is an alarming issue (8).

Smoking and physical inactivity are modifiable risk factors of CVD and premature death (9–11). Evidence reveals that even low-intensity tobacco use can be harmful to health (12). Multiple studies from China (13, 14), Japan (15), and South Korea (16) have shown that smoking may greatly increase the risk of premature death in hypertensive patients due to the synergistic effect between smoking and elevated blood pressure. In contrast, higher levels of physical activity can reduce the risk of all-cause mortality and CVD events in hypertensive patients (17, 18). Previous studies have established that the elderly and patients with various chronic diseases can obtain substantial health benefits through increasing physical activities (19–22).

Although relationship between smoking, physical activity and mortality has been well-established, the interaction of smoking and physical activity on mortality remains unclear. A good understanding of the interaction is important to identify target groups for interventions and to implement primary prevention strategies. Several studies have investigated the comprehensive influence of multiple health risk factors on health (23–27). However, most of these studies (24, 26) mainly focused on the association of the co-exposure of smoking and physical inactivity with adverse health outcomes, but failed to provide sufficient quantitative measures of interaction to explore the joint association in detail. Whether physical activity moderates the effect of smoking on mortality risk in the elderly hypertensive population has never been evaluated. Therefore, using data from a large-scale prospective cohort in China, our study aimed to explore the joint association of smoking and physical exercise with all-cause mortality and CVD mortality in elderly hypertensive patients. This research is very important in order to clarify whether there is an additional benefit of simultaneous interventions for smoking and lack of exercise.

## Methods

### Data source and study population

Shanghai Electronic Health Records Management System (EHR) holds information on electronic health records for Shanghai residents. Each patient has a unique personal identification number in this system. All institutions, including secondary and tertiary hospitals and primary healthcare centers, use EHR system to record clinical activities, regular follow-ups of primary health care, and collect information on physical examinations, chronic disease management and medication treatments (28, 29).

Since 2007, Minghang district of Shanghai has initiated primary care management for patients with hypertension (30). According to International Practical Guidelines for Hypertension (31), hypertension is defined as systolic blood pressure (SBP) ≥140 mmHg or diastolic blood pressure (DBP) ≥90 mmHg, or taking antihypertensive drugs. All patients diagnosed with hypertension are included in the primary care management. According to the standard operating procedures (SOPs) of EHR system, patients were required to have at least one physical examination every 6 months (28, 29).

Data collection, recording, and uploading were carried out following pre-established SOPs. According to the standardized information collection manual, primary medical staff was uniformly trained every year, and special supervisors were regularly assigned for supervision. To evaluate internal effectiveness of this registration system, a mid-term evaluation was conducted from February 1, 2008 to May 30, 2009. We randomly selected 1,700 hypertensive patients from EHR system, provided them with free physical examination and clinical consultation, and arranged 10 senior physicians to re-evaluate the patients. In the end, a total of 1,459 patients completed the reassessment. The consistent rates of diagnosis of hypertension, stroke, diabetes, left ventricular hypertrophy, atherosclerotic plaque and retinal artery stenosis were 0.97, 0.96, 0.93, 0.96, 0.79, and 0.83, respectively.

**Figure 1:**
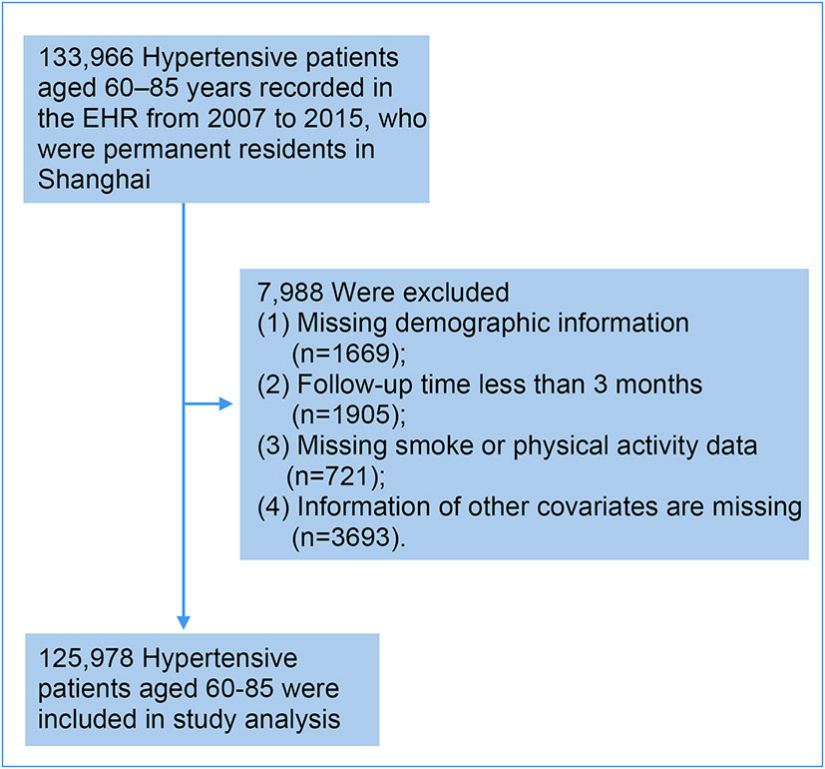
Study population. EHR system denotes the electronic health information system.

This study included all 133,966 hypertensive patients aged 60–85 years recorded in EHR from 2007 to 2015, who were permanent residents in Shanghai. Exclusion criteria were as follows: (1) Missing demographic information (*n* = 1669); (2) Follow-up time <3 months (*n* = 1905); (3) Missing smoke or physical activity data (*n* = 721); (4) Information of other covariates are missing (*n* = 3693). After exclusion, this study included 125,978 (94.0%) hypertensive patients aged 60–85 years [mean (SD) age, 70.5 (6.9) years] (Figure 1).

### Exposures

Self-reported smoking status and physical activity were obtained in face-to-face interviews. Smoking status was categorized into never-smoking and smoking. Never-smokers were defined as life-long non-smokers. Smokers included those who self-reported smoking regularly, occasionally and quit smoking at baseline.

Physical exercise was categorized into three groups (regular exercise, occasional exercise, and never exercise) (32). Regular exercise was defined as ≥150 min of moderate-intensity activities or ≥75 min of high-intensity activities per week. To be more specific, moderate-intensity activities referred to physical activities between 3 and 6 times the resting intensity, also equivalent to 50–60% of individual's physical capacity; high-intensity activities referred to physical activities more than six times the resting intensity, relative to 70–80% of individual's physical capacity. This definition of regular exercise was consistent with exercise intensity recommended by World Health Organization (WHO), which is applicable to the elderly (32). The exercise below this intensity was defined as occasional exercise.

### Outcomes

The outcomes of interest were all-cause mortality and CVD mortality. The primary cause of death was reported according to International Classification of Diseases, 10th Revision (ICD-10) (33), the corresponding code for cardiovascular disease was I00-I99. All death information has been verified by death documents provided by Minhang District Center for Disease Control and Prevention in Shanghai.

The follow-up in our study ended on 31 December 2018. The overall survival was defined as the time period between the day of patients firstly recorded until the day of outcome of interest, updating once every 3 months. The patient was censored on the date of the last follow-up in EHR.

### Covariates

Potential confounders were determined according to variable relationships shown in directed acyclic graph based on previous research (Supplementary Figure 1). We included gender, age, alcohol drinking (never drinking, occasional drinking, regular drinking defined as at least twice drinks per week for more than a year) (34), body mass index (BMI, calculated by weight in kilograms divided by the square of height in meters), diabetes (fasting blood glucose ≥7.0 mmol/L or 2-h blood glucose ≥11.1 mmol/L), classification of hypertension (grade 1 hypertension: SBP140-159 or DBP90-99, grade 2 hypertension: SBP160-179 or DBP100-109, grade 3 hypertension:SBP ≥ 180 or DBP ≥ 110) (31), family history of CVD (no, yes), family history of diabetes (no, yes), family history of hypertension (no, yes), and family history of stroke (no, yes).

### Statistical analysis

Basic characteristics of population according to different levels of smoking and physical activity were compared by chi-square test for categorical variables, and one-way analysis of variance or Kruskal-Wallis test for continuous variables where appropriate. Cumulative incidence of CVD mortality by smoking and exercise was estimated in the presence of competing risk, treating non-cardiovascular deaths as competing events (34, 35). Cumulative incidence of all-cause mortality was also presented.

Cox proportional hazards regression was used to estimate the individual and joint association of smoking and physical exercise on the risk of all-cause and CVD mortality. The proportional hazards assumption was evaluated by log-minus-log plots (35, 36), and the curves were approximately parallel as shown in Supplementary Figure 2.

We measured interactions on both additive and multiplicative scales. The coefficient of product term (physical exercise^*^smoking) in Cox regression could reflect the multiplicative interaction, which measures the relative change of risk (37). Three indicators were used to evaluate the additive interaction of smoking and never exercising, which measures the absolute change of risk: (1) Relative excess risk due to interaction (RERI), (2) attributable proportion due to interaction (AP), (3) synergy index (S), defined as follow (38);


$$\begin{array}{l} RERI=HR_{11}-HR_{10}-HR_{01}+1\\ AP=\frac{RERI}{HR_{11}}\\ S=\frac{HR_{11}-1}{\left(HR_{10}-1)+(HR_{01}-1\right)} \end{array}$$

Here *HR*_11_ represented the hazard ratio of people who smoke and never exercise, *HR*_10_ for people who smoke and exercise regularly, and *HR*_01_ for people who never smoke and never exercise. There was an additive interaction if RERI and AP were unequal to 0, or S was unequal to 1.

Stratified analysis was also presented to see whether the association of smoking with mortality modified by physical activity. Considering sex differences in smoking, the joint association of smoking and physical exercise were estimated by gender. And we calculated the joint association stratified by age to gain more specific information in elderly population. We also conducted interaction analyses using sex-stratified Cox regression model with age as time scale (39). Moreover, we excluded people with diabetes and family history of chronic diseases to assess if the relations were sensitive to comorbid chronic diseases and genetic factors. All analyses were two-sided test with a significant level of 0.05, performed by SAS 9.4 and Stata 16. Three measures of additive interaction were computing by SAS programs provided by Li and Chambless (40).

## Results

### Basic characteristics and mortality follow-up

Of 125,978 elderly hypertensive patients, the median age was 70.1 years old, smoking rate was 16.7%, and women accounted for 53.3%. Compared with never smokers, smokers were more likely to be male, younger, drink regularly, have more severe stage of hypertension and have family history of chronic diseases. Compared with those who never exercised, those who exercised regularly were more likely to be male, younger, have comorbid diabetes, have grade 3 hypertension and family history of chronic diseases (Table 1).

**Table 1:** Characteristics of participants according to physical activity and smoking (*n* = 125,978)^a^.

**Variables**	**Total**	**Physical activity**			**Smoke**	
		**Never**	**Occasional**	**Regular**	**No**	**Yes^b^**
		**(*N* = 39,350)**	**(*N* = 54,120)**	**(*N* = 32,508)**	**(*N* = 104,955)**	**(*N* = 21,023)**
**Gender**						
Male	58,891 (46.7)	16,528 (42.0)	25,835 (47.7)	16,528 (50.8)	38,612 (36.8)	20,279 (96.5)
Female	67,087 (53.3)	22,822 (58.0)	28,285 (52.3)	15,980 (49.2)	66,343 (63.2)	744 (3.5)
**Age (median, IQR^c^), years**	70.1 (11.8)	72.0 (12.6)	70.1 (11.7)	69.9 (10.6)	70.7 (11.9)	67.3 (10.6)
60–69	62,356 (49.5)	16,794 (42.7)	28,596 (52.8)	16,966 (52.2)	49,509 (47.2)	12,847 (61.1)
70–79	49,305 (39.1)	16,240 (41.3)	19,972 (36.9)	13,093 (40.3)	42,447 (40.4)	6,858 (32.6)
≥80	14,317 (11.4)	6,316 (16.1)	5,552 (10.3)	2,449 (7.5)	12,999 (12.4)	1,318 (6.3)
**BMI (median, IQR^c^), kg/m** ^**2**^	23.7 (3.8)	23.8 (4.1)	24.0 (3.8)	24.0 (3.7)	23.9 (3.9)	23.9 (3.7)
Underweight (<18.5 kg/m^2^)	4,098 (3.3)	1,634 (4.2)	1,611 (3.0)	853 (2.6)	3,465 (3.3)	633 (3.0)
Normal (18.5–24.9 kg/m^2^)	80,784 (64.1)	25,245 (64.2)	34,702 (64.1)	20,837 (64.1)	67,341 (64.2)	13,443 (63.9)
Overweight (25–29.9 kg/m^2^)	36,773 (29.2)	10,983 (27.9)	15,991 (29.5)	9,799 (30.1)	30,387 (29.0)	6,386 (30.4)
Obesity (≥30 kg/m^2^)	4,323 (3.4)	1,488 (3.8)	1,816 (3.4)	1,019 (3.1)	3,762 (3.6)	561 (2.7)
**Comorbid diabetes**	26,009 (20.6)	8,118 (20.6)	10,980 (20.3)	6,911 (21.3)	22,210 (21.2)	3,799 (18.1)
**Alcohol drinking**						
Never	104,572 (83.0)	34,318 (87.2)	43,761 (80.9)	26,493 (81.5)	95,049 (90.6)	9,523 (45.3)
Occasional	14,872 (11.8)	3,093 (7.9)	7,890 (14.6)	3,889 (12.0)	8,086 (7.7)	6,786 (32.3)
Regular	6,534 (5.2)	1,939 (4.9)	2,469 (4.6)	2,126 (6.5)	1,820 (1.7)	4,714 (22.4)
**Classification of Hypertension**						
Grade 1 hypertension	64,219 (51.0)	19,958 (50.7)	28,026 (51.8)	16,235 (49.9)	54,582 (52.0)	9,637 (45.8)
Grade 2 hypertension	42,909 (34.1)	13,329 (33.9)	18,582 (34.3)	10,998 (33.8)	35,275 (33.6)	7,634 (36.3)
Grade 3 hypertension	18,850 (15.0)	6,063 (15.4)	7,512 (13.9)	5,275 (16.2)	15,098 (14.4)	3,752 (17.8)
**Family history of CVD**						
No	123,118 (97.7)	38,510 (97.9)	53,023 (98.0)	31,585 (97.2)	102,624 (97.8)	20,494 (97.5)
Yes	2,860 (2.3)	840 (2.1)	1,097 (2.0)	923 (2.8)	2,331 (2.2)	529 (2.5)
**Family history of diabetes**						
No	120,830 (95.9)	37,880 (96.3)	51,920 (95.9)	31,030 (95.5)	100,811 (96.1)	20,019 (95.2)
Yes	5,148 (4.1)	1,470 (3.7)	2,200 (4.1)	1,478 (4.5)	4,144 (3.9)	1,004 (4.8)
**Family history of hypertension**						
No	83,006 (65.9)	27,215 (69.2)	35,636 (65.8)	20,155 (62.0)	70,314 (67.0)	12,692 (60.4)
Yes	42,972 (34.1)	12,135 (30.8)	18,484 (34.2)	12,353 (38.0)	34,641 (33.0)	8,331 (39.6)
**Family history of stroke**						
No	123,415 (98.0)	38,569 (98.0)	53,130 (98.2)	31,716 (97.6)	103,010 (98.1)	20,405 (97.1)
Yes	2,563 (2.0)	781 (2.0)	990 (1.8)	792 (2.4)	1,945 (1.9)	618 (2.9)

a Values are n (%) unless explained otherwise; CVD, cardiovascular disease.

The chi-square test was used to compare categorical variables and the Kruskal-Wallis test was used to compare continuous variables, and the P-values were < 0.01 between all groups, except BMI for smoker and non-smoker (P = 0.68).

b Smoking, including quitting.

c IQR, interquartile range calculated by Q3-Q1.

**Figure 2:**
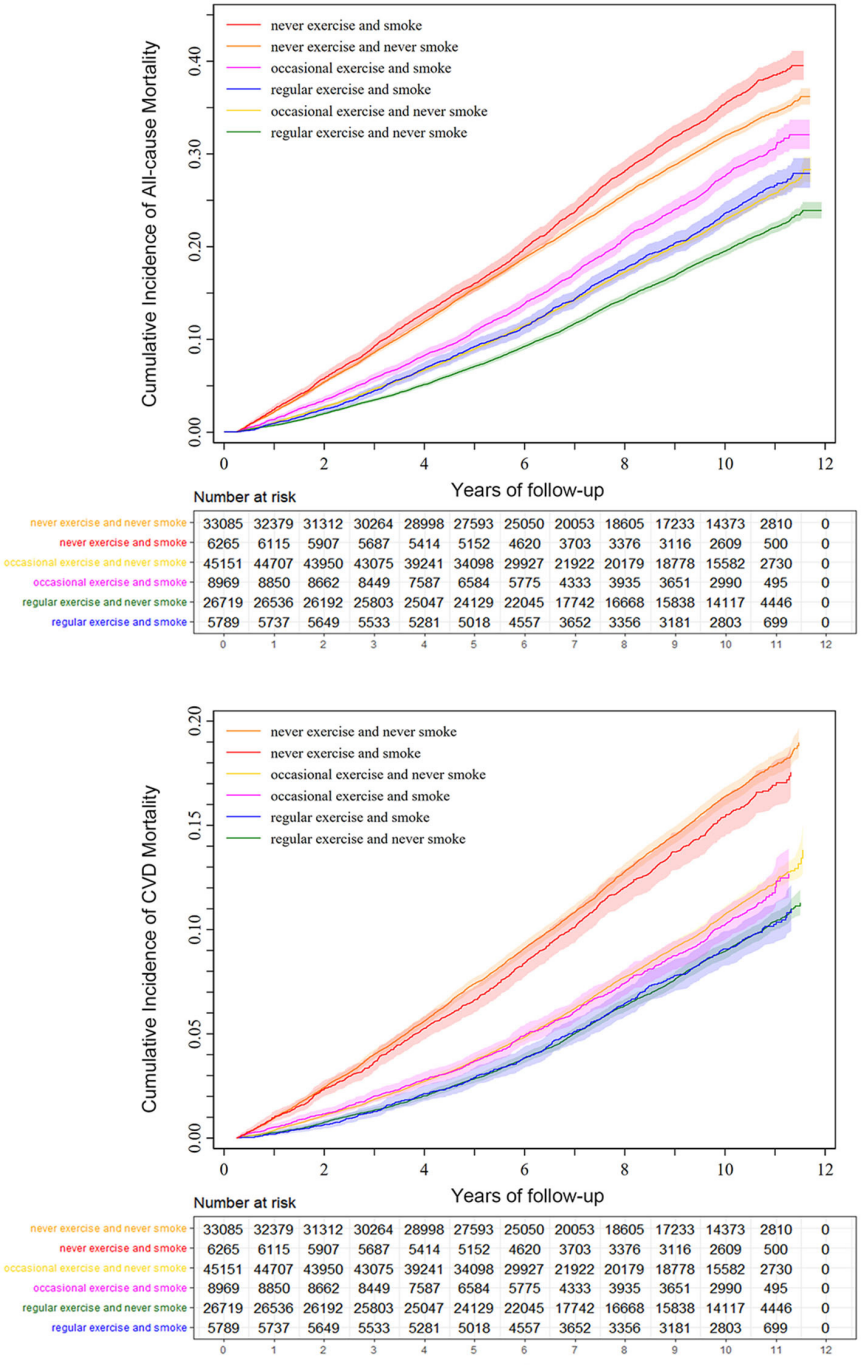
Cumulative incidence and corresponding 95% CI of all-cause and CVD mortality by smoke and exercise. Estimation of CVD mortality accounted for competing risk treating non-cardiovascular deaths as competing events.

During the 1,004,801 person-years of follow-up (median follow-up: 8.9 years, interquartile range: 5.8–10.6 years), 28,250 deaths from all causes and 13,164 deaths from CVD were observed. Overall, people with lower intensity of physical activity had a higher mortality rate (**Table 3**). Cumulative incidence estimation according to smoke and exercise showed similar results (Figure 2).

### Individual associations of smoking and physical activity

Table 2 presented the independent associations of smoking and physical activity on mortality risk. Higher levels of physical activity and never smoking were associated with reduced risk of death. Compared with never exercise, regular exercise was associated with lower risk of all-cause mortality by 36% (HR: 0.64, 95% CI: 0.62–0.66) and CVD mortality by 40% (HR: 0.60, 95% CI: 0.57–0.63). Compared with smokers, non-smokers had lower hazard ratios for all-cause death (HR: 0.73, 95%CI: 0.71–0.76) and CVD death (HR: 0.77, 95% CI: 0.73–0.82).

**Table 2:** The hazard ratios for the independent associations of smoking and physical activity on mortality risk^a^.

	**Person-years of follow-up**	**All-cause mortality**			**CVD mortality**		
		**Deaths**	**Rate /1,000 person-years**	**HR^b^ (95% CI)**	**Deaths**	**Rate /1,000 person-years**	**HR^b^ (95% CI)**
**Physical activity**							
Never	317,050	12,041	37.98	1.00	5,993	18.90	1.00
Occasional	405,809	10,085	24.85	0.76 (0.74, 0.78)	4,457	10.98	0.70 (0.67, 0.73)
Regular	281,942	6,124	21.72	0.64 (0.62, 0.66)	2,714	9.63	0.60 (0.57, 0.63)
**Smoke**							
Yes (including quitting)	164,805	5,281	32.04	1.00	2,102	12.75	1.00
No	839,997	22,969	27.34	0.73 (0.71, 0.76)	11,062	13.17	0.77 (0.73, 0.82)

a CI, confidence interval; CVD, cardiovascular disease.

b Adjusted for age, gender, alcohol drinking, BMI, comorbid diabetes, classification of hypertension, family history of CVD, family history of diabetes, family history of hypertension, family history of stroke, and mutually for either smoking or physical activity; the product term (physical activity ^*^ smoke) was not statistically significant in the models, so it was not included in the independent effect estimates.

### Joint associations of smoking and physical activity

Table 3 shows the joint association of smoking and physical activity. In both smokers and non-smokers, lower exercise levels were associated with higher risk of death, but the strength of this adverse association was stronger among smokers. Taking both regular exercise and non-smoking as reference group, risk of death increased as concurrent exposures to smoking and lacking exercise increased. Smokers who never exercised had greatest risk for mortality (HR for All-cause: 2.10, 95% CI: 1.99–2.21; HR for CVD: 2.19, 95% CI: 2.02–2.38), but increasing physical activity levels might counteract some of these extra risks. Similar patterns were observed for all-cause mortality and CVD mortality by different combinations of smoking and physical activity (Table 3). We found a significant additive interaction between smoking and never exercise, with RERI, AP, and S being 0.29 (95% CI: 0.10–0.49), 0.13 (0.05–0.22) and 1.33 (1.10–1.61) for CVD mortality, and 0.20 (0.08–0.33), 0.10 (0.04–0.15) and 1.23 (1.08–1.39) for all-cause mortality, respectively. However, the multiplicative interaction was not statistically significant (all-cause: 1.00, 0.96–1.04; CVD: 0.97, 0.91–1.03).

**Table 3:** Joint association of smoking and physical activity with mortality risk^a^.

**Measurements**		**Number**	**Person-years of follow-up (average person-years)**	**All-cause mortality**		**CVD mortality**	
				**Deaths (Rate /1,000 person-years)**	**Hazard ratio (95% CI)**	**Deaths (Rate /1,000 person-years)**	**Hazard ratio (95% CI)**
**Joint association**						
Never smoke	Regular exercise	26,719	233,231 (8.7)	4,875 (20.90)	1.00	2,240 (9.60)	1.00
	Occasional exercise	45,151	339,442 (7.5)	8,133 (23.96)	1.17 (1.13, 1.21)	3,734 (11.00)	1.16 (1.10, 1.22)
	Never exercise	33,085	267,324 (8.1)	9,961 (37.26)	1.55 (1.50, 1.61)	5,088 (19.03)	1.65 (1.57, 1.74)
Smoke (include quitting)	Regular exercise	5,789	48,712 (8.4)	1,249 (25.64)	1.34 (1.25, 1.43)	474 (9.73)	1.24 (1.12, 1.38)
	Occasional exercise	8,969	66,367 (7.4)	1,952 (29.41)	1.64 (1.55, 1.73)	723 (10.89)	1.49 (1.36, 1.63)
	Never exercise	6,265	49,726 (7.9)	2,080 (41.83)	2.10 (1.99, 2.21)	905 (18.20)	2.19 (2.02, 2.38)
**Interaction on additive scale^b^**					**Estimates (95% CI)**		**Estimates (95% CI)**
RERI					0.20 (0.08, 0.33)		0.29 (0.10, 0.49)
AP					0.10 (0.04, 0.15)		0.13 (0.05, 0.22)
S					1.23 (1.08, 1.39)		1.33 (1.10, 1.61)
***P*** **-value**					0.001		0.003
**Interaction on multiplicative scale**					**Estimates (95% CI)**		**Estimates (95% CI)**
Physical activity * Smoke					1.00 (0.96, 1.04)		0.97 (0.91, 1.03)
***P*** **-value**					0.96		0.28

a Models adjusted for age, gender, alcohol drinking, BMI, comorbid diabetes, classification of hypertension, family history of CVD, family history of diabetes, family history of hypertension, family history of stroke, and the product term (physical activity ^*^ smoke).

b RERI, relative excess risk due to interaction; AP, attributable proportion due to interaction; S, synergy index; Additive interactions exist if RERI and AP are unequal to 0, or S is unequal to 1.

### Stratified analyses

Non-smokers had lower mortality risk than smokers among different levels of physical activity, and this beneficial association became stronger in people who exercised regularly (Supplementary Table 1). People who had higher intensity of exercise were associated with lower mortality risk than people who never exercised despite varying levels of smoking (Supplementary Table 2).

Due to the large gender differences in smoking rates (34.44% in males and 1.11% in females), we calculated joint associations of smoking and exercise according to different genders. In men and women, the pattern of combined effects was consistent with that in entire population, but estimates were greater in men (Table 4). Results in different age groups were also similar. The joint association of physical activity and smoking on mortality were also found in individuals aged 80 years and above (Supplementary Table 3).

**Table 4:** The joint association of smoking and physical activity on mortality stratified by gender^a^.

**Joint association of different exposure combinations**		**Male (*****n*** = **99,034)**		**Female (*****n*** = **113,348)**	
		**Deaths (rate/1,000 person-years)**	**HR (95% CI)**	**Deaths (rate/1,000 person-years)**	**HR (95% CI)**
**All-cause mortality**				
Never smoke	Regular exercise	2,365 (25.43)	1.00	2,510 (17.90)	1.00
	Occasional exercise	3,616 (29.16)	1.20 (1.14, 1.26)	4,517 (20.97)	1.12 (1.07, 1.18)
	Never exercise	3,737 (46.41)	1.69 (1.60, 1.77)	6,224 (33.32)	1.44 (1.37, 1.51)
Smoke (including quitting)	Regular exercise	1,210 (25.65)	1.37 (1.28, 1.47)	39 (25.45)	1.27 (0.92, 1.75)
	Occasional exercise	1,895 (29.50)	1.69 (1.58, 1.79)	57 (26.65)	1.27 (0.98, 1.66)
	Never exercise	1,986 (41.62)	2.16 (2.03, 2.30)	94 (46.90)	1.78 (1.45, 2.19)
**CVD mortality**				
Never smoke	Regular exercise	1,000 (10.75)	1.00	1,240 (8.84)	1.00
	Occasional exercise	1,544 (12.45)	1.23 (1.13, 1.33)	2,190 (10.17)	1.10 (1.02, 1.17)
	Never exercise	1,781 (22.12)	1.89 (1.75, 2.04)	3,307 (17.70)	1.47 (1.38, 1.57)
Smoke (including quitting)	Regular exercise	452 (9.58)	1.29 (1.15, 1.45)	22 (14.36)	1.41 (0.92, 2.17)
	Occasional exercise	697 (10.85)	1.57 (1.42, 1.74)	26 (12.16)	1.12 (0.76, 1.66)
	Never exercise	859 (18.00)	2.34 (2.13, 2.57)	46 (22.95)	1.67 (1.24, 2.25)

a Models adjusted for age, alcohol drinking, BMI, comorbid diabetes, classification of hypertension, family history of CVD, family history of diabetes, family history of hypertension, family history of stroke, and the product term (physical activity ^*^ smoke).

### Sensitivity analyses

The results of interaction analyses using sex-stratified Cox regression model with age as time scale were similar to the primary analyses (Supplementary Table 4). Analyses excluding people with diabetes and family history of chronic diseases did not change the results (Supplementary Table 5).

## Discussion

We found that regular exercise and never smoking were associated with lower risk of all-cause mortality and CVD mortality in an elderly hypertensive population. There was an additive interaction between smoking and physical inactivity, with the concurrence of both risk factors was associated with more than 2-fold risk of death [HR: all-cause 2.10 (1.99–2.21), CVD 2.19 (2.02–2.38)]. The estimates were more pronounced in men than in women. Smokers who never exercised had the greatest risk for mortality among all exposure groups, but increasing physical activity levels might counteract some of the extra risks. Although multiplicative interaction was not significant, we found a tendency that the strength of beneficial association of non-smoking became stronger among regular exerciser in stratified analyses.

Additive interaction measures the absolute change of risk, while multiplicative interaction measures the relative change of risk. Additive interaction has more public health significance and is more related to biological interaction (41–43). When health resources are limited, it is recommended to make decisions based on additive interaction to maximize the benefits of intervention (41–43).

Most of the available studies, yet not all, supported a joint association of smoking and physical activity with mortality. In US adults, combined effect of smoking and physical inactivity could significantly advance death by at least 2.4 years, which was consistent with our study (24). Similarly, a cohort study from Finland showed that risk of death was higher among inactive smokers (HR: 3.27, 95% CI: 2.05–5.22), compared with vigorously active non-smokers (26). A study among white-collar workers in the UK showed a lower incidence of coronary heart disease among non-smokers who engage in vigorous physical activity (4.2%) compared with smokers reporting no activity (11.5%) (44). However, study by Rehm et al. (45) indicated no interaction between smoking and exercise, but their calculated indicators of interaction were insufficient, using only the Mantel-Haenszel test (*p* > 0.1); the study by O'Donovan et al. (46) measured additive and multiplicative interactions between physical activity and smoking in-depth based on surveillance data of UK households and found a tendency of additive interaction, but the results were statistically insignificant. Our study show that smoking and physical inactivity interact on an additive scale. From the perspective of public health, this means that prevention of either smoking or physical inactivity not only reduces the risk of mortality by eliminating the independent effect of this factor, but also prevents cases caused by the interaction of these two factors. Health care providers should ensure that recommendation to promote physical activity and long-term non-smoking are addressed as part of routine care. If one already had poor behavior habits, early change of these habits could be salutary (47).

However, tobacco control in China faced substantial barriers due to low willingness of smoking cessation (48). China is the largest consumer of tobacco worldwide. In 2018, China Adult Tobacco Survey Report showed that smoking rate in China was 26.6%, and the rate among men was 50.5% (49). At present, smokers' willingness to quit smoking in China (16.1%) (49) was much lower than those in other middle and high-income countries (Canada 81.1%, Australia 75.6%, the UK 65.3%, the US 75.1%) (50). Based on the findings of our study, we can invest more efforts in promoting physical activity as a way to reduce mortality risk among smokers.

The following mechanisms may underlie the interaction between smoking and physical activity: (1) Smoking may increase the risk of CVD and premature deaths by elevating blood lipid and oxidative stress levels, while exercise may help reduce post-prandial oxidative stress in smokers by increasing the activity of endogenous antioxidant enzymes and by improving clearance of blood triglyceride and glucose (51). (2) Increased level of physical activity may help alleviate the withdrawal symptoms in smokers and increase the success rate of quitting (52, 53). Quitting smoking would cause weight gain, which can reduce some people's motivation to quit, especially women. And exercise can reduce weight gain (54, 55), activity restriction, pain, depression, and anxiety after quitting (56). (3) Smoking may affect lung function and have a negative impact on exercise capacity (57).

The main strength of this study is the large representative study sample that including all hypertensive patients aged over 60 years in Minhang District, Shanghai. In Shanghai, individuals aged over 35 years would be asked to measure blood pressure at their first medical appointment, which means we can identify majority of hypertensive patients (58). No previous study has examined the interaction between smoking and physical activity in-depth in hypertensive population or aging population, and this study provides research evidence on these issues using prospective data.

This study also has some limitations. Firstly, data of smoking and physical activity were obtained based on register system thus the intensity and duration of exposures might be crude, and we only measured exposures at baseline while they can change over time; however, such misclassification may have biased the associations toward null, which means we may have underestimated the individual and joint associations of smoking and physical activity. Secondly, the measuring of smoking and physical activity was self-reported, which may be subject to recall bias. However, we classified them as dichotomous or trichotomous variables, which may reduce recall bias to some extent. Thirdly, this study was unable to control for education background and all comorbidities that could be confounders, some diseases may limit exercise ability and also be risk factors for death, such as heart failure and stroke. But we controlled for family history of chronic diseases including CVD and stroke, and sensitivity analyses excluding people with diabetes and family history of chronic diseases yielded similar results. Future studies could quantitatively measure the exposures and regularly monitor their changes to obtain more accurate estimates. Subsequent studies can monitor the physical activity level using an accelerometer and define the smoke level with a more adequate classification. It is also a suggestion to see if there are differences between different types of physical activities.

## Conclusion and clinical implications

Our study suggested that smoking and physical inactivity together may have amplified association on death compared to their independent associations, highlighting the importance of improving behavioral factors in combination. In the elderly hypertensive population, smokers who never exercised had the greatest risk for mortality among all exposure groups, but increasing physical activity levels might counteract some of the extra risks. Clinically, physicians should be aware of this increased risk among smokers who never exercised. Health care providers should ensure that recommendation to promote physical activity and long-term non-smoking are addressed as part of routine care, which may help to prevent cases caused by the interplay of these two factors. Government should promote primary prevention strategies that improve behavioral factors in combination and promoting a comprehensive healthy lifestyle in hypertensive elderly. And government could invest more efforts in promoting physical activity as a way to reduce mortality risk among smokers when tobacco control faced substantial barriers.

## Data availability statement

The original contributions presented in the study are included in the article/Supplementary materials, further inquiries can be directed to the corresponding authors.

## Ethics statement

The studies involving human participants were reviewed and approved by the Institutional Review Board of Center for Disease Control and Prevention in Minhang District, Shanghai (No: EC-P-2019-009). Written informed consent for participation was not required for this study in accordance with the national legislation and the institutional requirements.

## Author contributions

YYu, GQ, and HX: had full access to all of the data in the study, take responsibility for the integrity of the data, and the accuracy of the data analysis. YYu, YH, and GQ: study concept and design and study supervision. YYa: drafting of the manuscript. YYu, JL, XiaL, and ZL: critical revision of the manuscript for important intellectual content. YYa, HX, YH, GQ, and YYu: statistical analysis. YYu, JL, GQ, YX, and XinL: obtained funding. HX, YH, GQ, YYu, and JL: administrative, technical, or material support. All authors acquisition, analysis, or interpretation of data. All authors contributed to the article and approved the submitted version.

## Funding

This study was supported by a grant from Shanghai Rising-Star Program (21QA1401300) to YYu, a grant from Shanghai Municipal Natural Science Foundation (22ZR1414900) to YYu, grants from the Independent Research Fund Denmark (DFF-6110- 00019B, DFF-9039-00010B, and DFF-1030-00012B) to JL, a grant from the Nordic Cancer Union (R275-A15770) to JL, a grant from the Karen Elise Jensens Fond (2016) to JL, a grant from Novo Nordisk Foundation (NNF18OC0052029) to JL, grants from the National Natural Science Foundation of China (82073570 and 11871164) to GQ, Shanghai Municipal Science and Technology Major Project (ZD2021CY001) to GQ, Three-year Action Program of Shanghai Municipality for Strengthening the Construction of Public Health System (GWV-10.1-XK05) Big Data and Artificial Intelligence Application to GQ, Shanghai Municipal Nature Science Foundation (19ZR1445900), Min hang district key disciplines in public health (MGWXK01) and Health Consortium Foundation of Fudan University and Minhang District Health Committee (2019FM02) to HX. XiaL is supported by the European Union's Horizon 2020 Research and Innovation Programme under the Marie Sklodowska-Curie grant agreement No. 891079. The funders had no role in study design, data collection and analysis, decision to publish, or preparation of the manuscript.

## Conflict of interest

The authors declare that the research was conducted in the absence of any commercial or financial relationships that could be construed as a potential conflict of interest.

## Publisher's note

All claims expressed in this article are solely those of the authors and do not necessarily represent those of their affiliated organizations, or those of the publisher, the editors and the reviewers. Any product that may be evaluated in this article, or claim that may be made by its manufacturer, is not guaranteed or endorsed by the publisher.
