# Supplementary material for: Joint association of smoking and physical activity with mortality in elderly hypertensive patients: A Chinese population-based cohort study in 2007–2018
Source: Front Public Health. 2022 Sep 29;10:1005260. doi: 10.3389/fpubh.2022.1005260 (PMC9558130; doi:10.3389/fpubh.2022.1005260)
Supplement: Supplementary file 1 [file Data_Sheet_1.docx]

**Supplements**

**Joint association of smoking and physical activity with mortality in elderly hypertensive patients: a Chinese population-based cohort study in 2007-2018**

eFigure 1. Causal diagrams showing the relationship between variables

eFigure 2. Test of Cox proportional hazards assumption of physical activity and smoking

eTable 1. Association between smoking and mortality stratified by physical activity

eTable 2. Association between physical activity and mortality stratified by smoking

eTable 3. The joint association of smoking and physical activity on mortality stratified by age

eTable 4. Sensitivity analysis using sex-stratified Cox regression model with age as time scale.

eTable 5. Sensitivity analysis excluded people with diabetes and family history of chronic diseases (n= 64871).


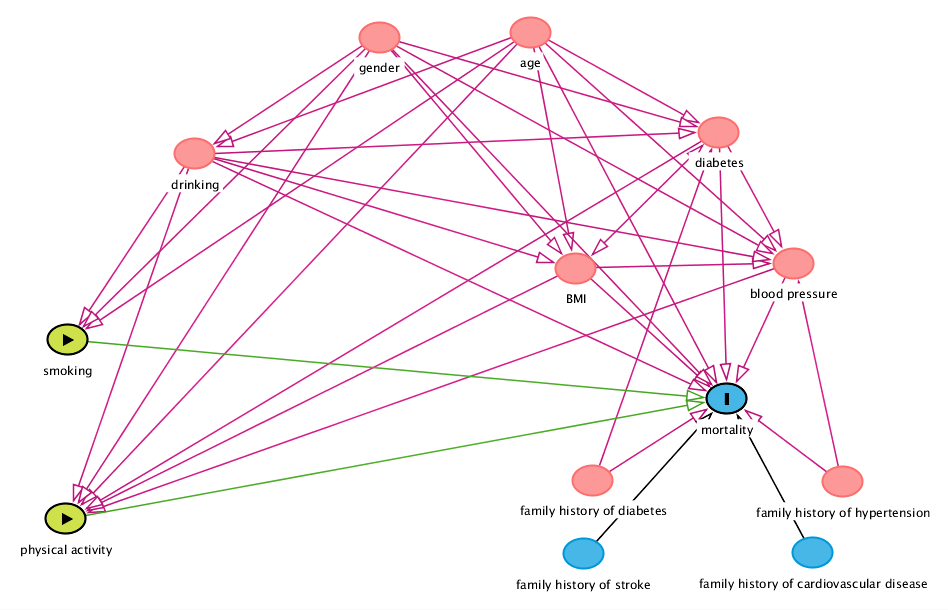


eFigure 1. Causal diagrams showing the relationship between variables


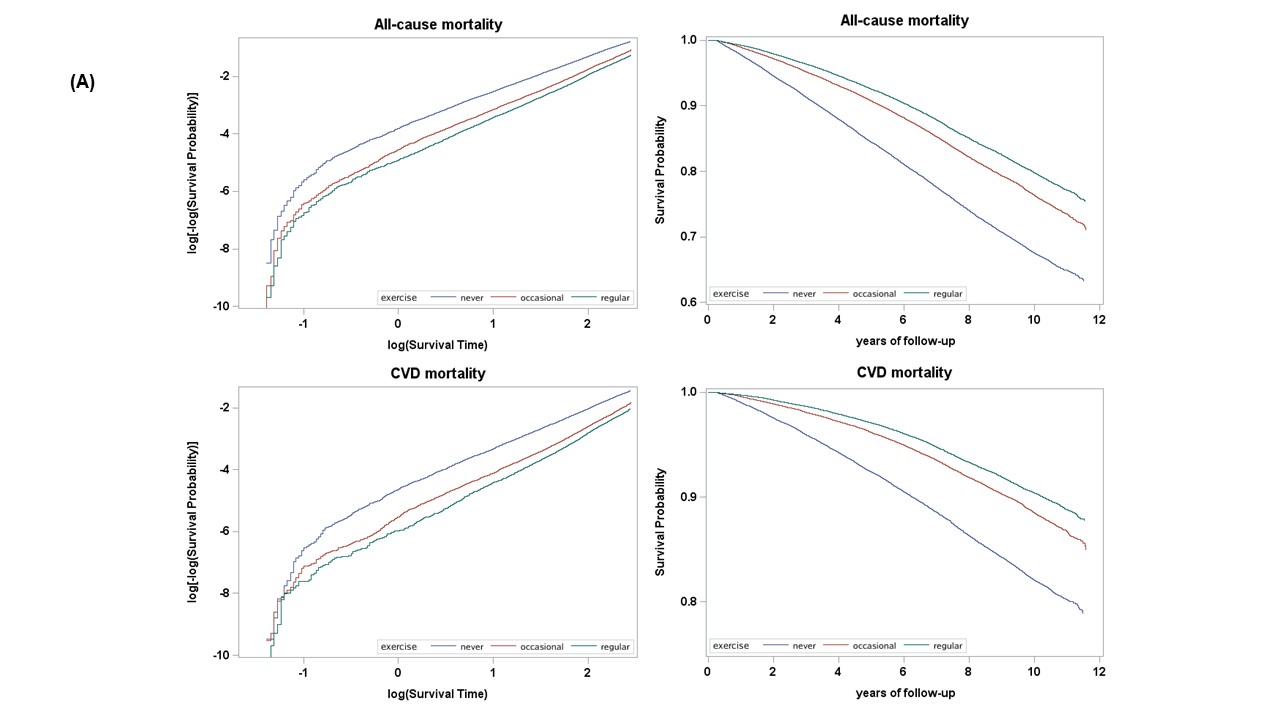


eFigure 2 (A). Test of Cox proportional hazards assumption of physical activity


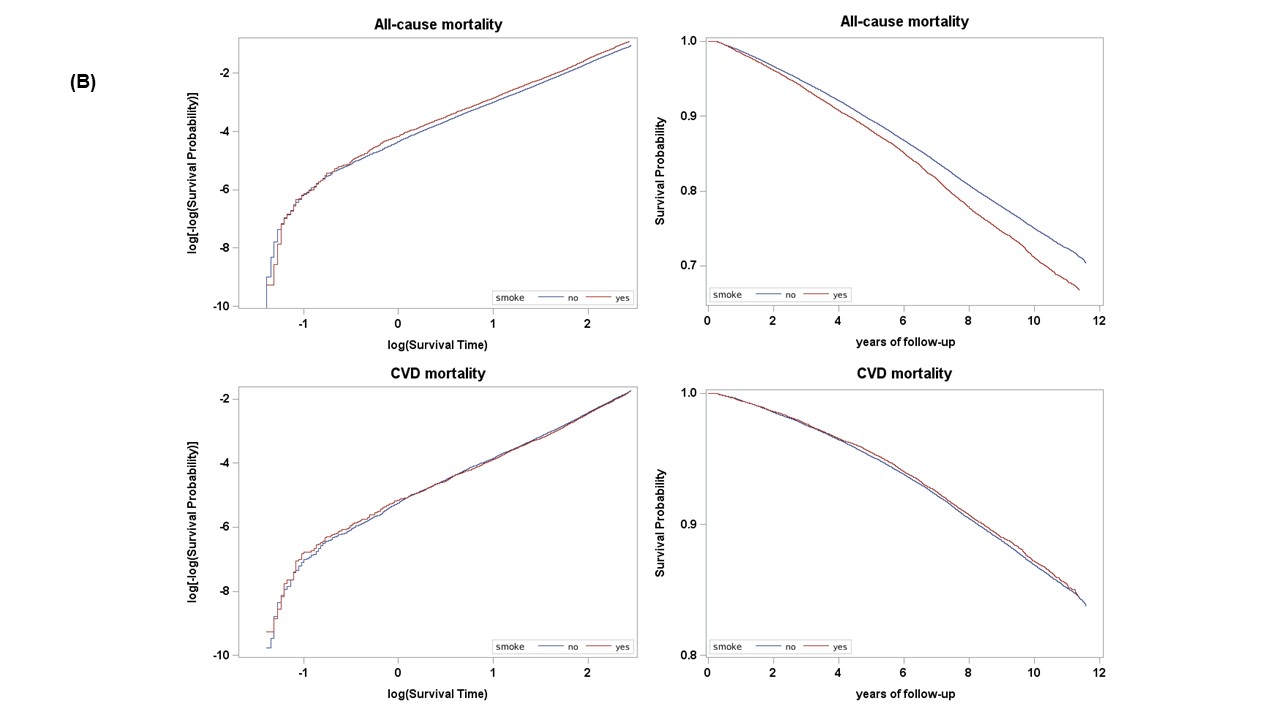


eFigure 2 (B). Test of Cox proportional hazards assumption of smoking

eFigure 2. Test of Cox proportional hazards assumption of physical activity and smoking

**Stratified analyses:**

**1. Stratified by physical activity:**

| eTable 1. Association between smoking and mortality stratified by physical activity ^a^. | | | | | |
| --- | --- | --- | --- | --- | --- |
| **Physical activity** | **Smoke** | **All-cause mortality** | | **CVD mortality** | |
|  |  | **Deaths (Rate /1000 person-years)** | **HR (95% CI)** | **Deaths (Rate /1000 person-years)** | **HR (95% CI)** |
| **Regular (n=50535)** | **Yes^b^** | 4,875 (20.90) | 1.00 | 2,240 (9.60) | 1.00 |
|  | **No** | 1,249 (25.64) | 0.71(0.66,0.76) | 474 (9.73) | 0.73(0.65,0.81) |
| **Occasional (n=93464)** | **Yes^b^** | 8,133 (23.96) | 1.00 | 3,734 (11.00) | 1.00 |
|  | **No** | 1,952 (29.41) | 0.71(0.67,0.76) | 723 (10.89) | 0.78(0.71,0.86) |
| **Never (n=68383)** | **Yes^b^** | 9,961 (37.26) | 1.00 | 5,088 (19.03) | 1.00 |
|  | **No** | 2,080 (41.83) | 0.77(0.72,0.81) | 905 (18.20) | 0.80(0.74,0.87) |
| ^a^ Models adjusted for age, gender, alcohol drinking, BMI, comorbid diabetes, classification of hypertension, family history of CVD, family history of diabetes, family history of hypertension, and family history of stroke.  ^b^ Smoking, including quitting. | | | | | |

**2. Stratified by smoke:**

| eTable 2. Association between physical activity and mortality stratified by smoking ^a^. | | | | | |
| --- | --- | --- | --- | --- | --- |
| **Smoke** | **Physical activity** | **All-cause mortality** | | **CVD mortality** | |
|  |  | **Deaths (Rate /1000 person-years)** | **HR (95% CI)** | **Deaths (Rate /1000 person-years)** | **HR (95% CI)** |
| **No**  **(n=22,969)** | Never | 9,961 (37.26) | 1.00 | 5,088 (19.03) | 1.00 |
|  | Occasional | 8,133 (23.96) | 0.75 (0.73,0.77) | 3,734 (11.00) | 0.71 (0.68,0.74) |
|  | Regular | 4,875 (20.90) | 0.65 (0.62,0.67) | 2,240 (9.60) | 0.61 (0.58,0.64) |
| **Yes (including quitting, n=5,281)** | Never | 2,080 (41.83) | 1.00 | 905 (18.20) | 1.00 |
|  | Occasional | 1,952 (29.41) | 0.78 (0.73,0.83) | 723 (10.89) | 0.67 (0.61,0.74) |
|  | Regular | 1,249 (25.64) | 0.64 (0.59,0.68) | 474 (9.73) | 0.56 (0.50,0.63) |
| ^a^ Models adjusted for age, gender, alcohol drinking, BMI, comorbid diabetes, classification of hypertension, family history of CVD, family history of diabetes, family history of hypertension, and family history of stroke. | | | | | |

**3. Stratified by age:**

| eTable 3. The joint association of smoking and physical activity on mortality stratified by age ^a^. | | | | | | | |
| --- | --- | --- | --- | --- | --- | --- | --- |
| **Joint** **association of different exposure combinations** | | **60-69 (n=62356)** | | **70-79 (n=49305)** | | **≥80 (n=14317)** | |
|  |  | **Deaths (Rate/1000 person-years)** | **HR (95% CI)** | **Deaths (Rate/1000 person-years)** | **HR (95% CI)** | **Deaths (Rate/1000 person-years)** | **HR (95% CI)** |
| **All-cause mortality** | |  |  |  |  |  |  |
| Never smoke | Regular exercise | 980(8.21) | 1.00 | 2825(28.86) | 1.00 | 1070(66.77) | 1.00 |
|  | Occasional exercise | 1533(8.70) | 1.13(1.04,1.23) | 4363(33.18) | 1.23(1.17,1.29) | 2237(70.48) | 1.13(1.05,1.22) |
|  | Never exercise | 1457(12.35) | 1.54(1.42,1.67) | 5027(44.51) | 1.64(1.57,1.72) | 3477(95.53) | 1.55(1.45,1.66) |
| Smoke^b^ | Regular exercise | 430(14.12) | 1.35(1.20,1.52) | 662(39.81) | 1.25(1.14,1.36) | 157(96.47) | 1.34(1.13,1.59) |
|  | Occasional exercise | 623(14.52) | 1.48(1.33,1.65) | 1019(49.63) | 1.63(1.51,1.75) | 310(105.65) | 1.56(1.37,1.78) |
|  | Never exercise | 638(20.76) | 1.99(1.79,2.22) | 1016(63.41) | 2.03(1.88,2.18) | 426(143.39) | 2.10(1.87,2.36) |
| **CVD mortality** | |  |  |  |  |  |  |
| Never smoke | Regular exercise | 351(2.94) | 1.00 | 1300(13.28) | 1.00 | 589(36.76) | 1.00 |
|  | Occasional exercise | 536(3.04) | 1.14(1.00,1.30) | 2022(15.37) | 1.25(1.16,1.34) | 1176(37.05) | 1.09(0.99,1.20) |
|  | Never exercise | 546(4.63) | 1.62(1.41,1.85) | 2590(22.93) | 1.83(1.71,1.95) | 1952(53.63) | 1.55(1.41,1.70) |
| Smoke^b^ | Regular exercise | 143(4.70) | 1.28(1.04,1.57) | 266(16.00) | 1.17(1.02,1.34) | 65(39.94) | 1.10(0.85,1.43) |
|  | Occasional exercise | 195(4.55) | 1.36(1.13,1.64) | 404(19.68) | 1.52(1.35,1.71) | 124(42.26) | 1.24(1.02,1.52) |
|  | Never exercise | 239(7.78) | 2.14(1.79,2.55) | 458(28.58) | 2.15(1.92,2.40) | 208(70.01) | 2.03(1.72,2.39) |
| ^a^ Models adjusted for gender, alcohol drinking, BMI, comorbid diabetes, classification of hypertension, family history of CVD, family history of diabetes, family history of hypertension, family history of stroke, and the product term(physical activity * smoke).  ^b^ Smoke, including quitting. | | | | | | | |

**Sensitivity analysis：**

| eTable 4. Sensitivity analysis using sex-stratified Cox regression model with age as time scale. ^a^ | | | |
| --- | --- | --- | --- |
| **Estimates (95% CI)** | | **All-cause mortality** | **CVD mortality** |
| **Joint association of different exposure combinations** | | |  |
| Never smoke | Regular exercise | 1.00 | 1.00 |
|  | Occasional exercise | 1.17(1.13,1.22) | 1.16(1.10,1.22) |
|  | Never exercise | 1.55(1.50,1.61) | 1.64(1.56,1.72) |
| Smoke (including quitting) | Regular exercise | 1.32(1.23,1.40) | 1.21(1.09,1.34) |
|  | Occasional exercise | 1.61(1.53,1.71) | 1.45(1.32,1.58) |
|  | Never exercise | 2.07(1.96,2.19) | 2.15(1.98,2.33) |
| **Interaction on additive scale ^b^** | |  |  |
| RERI | | 0.20 (0.08,0.32) | 0.29 (0.10,0.48) |
| AP | | 0.10 (0.04,0.15) | 0.14 (0.06,0.22) |
| S | | 1.23 (1.08,1.40) | 1.34 (1.11,1.64) |
| ***P*-value** | | 0.001 | 0.002 |
| **Interaction on multiplicative scale** | |  |  |
| Physical activity * Smoke | | 1.00 (0.96,1.04) | 0.96 (0.90, 1.02) |
| ***P*-value** | | 0.89 | 0.18 |
| ^a^ Estimates were calculated using sex-stratified Cox regression models, with age as time scale. Models adjusted for alcohol drinking, BMI, comorbid diabetes, classification of hypertension, family history of CVD, family history of diabetes, family history of hypertension, family history of stroke, and the product term(physical activity * smoke).  ^b^ Additive interactions exist if RERI and AP are not equal to 0, or S is not equal to 1. | | | |

| eTable 5. Sensitivity analysis excluded people with diabetes and family history of chronic diseases (n= 64871). ^a^ | | | |
| --- | --- | --- | --- |
| **Estimates (95% CI)** | | **All-cause mortality** | **CVD mortality** |
| **Joint association of different exposure combinations** | | |  |
| Never smoke | Regular exercise | 1.00 | 1.00 |
|  | Occasional exercise | 1.18 (1.13,1.24) | 1.16 (1.08,1.25) |
|  | Never exercise | 1.54 (1.47,1.62) | 1.43 (1.27,1.61) |
| Smoke (including quitting) | Regular exercise | 1.41 (1.30,1.54) | 1.27 (1.10,1.46) |
|  | Occasional exercise | 1.69 (1.56,1.82) | 1.62 (1.51,1.73) |
|  | Never exercise | 2.21 (2.06,2.38) | 2.20 (1.97,2.45) |
| **Interaction on additive scale ^b^** | |  |  |
| RERI | | 0.26 (0.08,0.43) | 0.31 (0.05,0.56) |
| AP | | 0.12 (0.04,0.19) | 0.14 (0.03,0.25) |
| S | | 1.27 (1.07,1.49) | 1.35 (1.04,1.75) |
| ***P*-value** | | 0.004 | 0.019 |
| **Interaction on multiplicative scale** | |  |  |
| Physical activity * Smoke | | 0.99 (0.94,1.05) | 0.96 (0.88,1.04) |
| ***P*-value** | | 0.80 | 0.30 |
| ^a^ Models adjusted for age, gender, alcohol drinking, BMI, classification of hypertension, and the product term(physical activity * smoke).  ^b^ Additive interactions exist if RERI and AP are not equal to 0, or S is not equal to 1. | | | |
